# Monochorionic twins discordant for trisomy 13: A case report, systematic literature search and synthesis of available evidence

**DOI:** 10.1002/ccr3.3244

**Published:** 2020-09-01

**Authors:** Emily F. Cornish, Ruwan Wimalasundera, George Attilakos

**Affiliations:** ^1^ UCL Elizabeth Garrett Anderson Institute for Women’s Health University College London (UCL) London UK; ^2^ Fetal Medicine Unit University College London Hospitals London UK

**Keywords:** amniocentesis, discordant anomalies, monochorionic twins, selective termination, trisomy 13

## Abstract

This article presents the tenth reported case of monochorionic twins discordant for trisomy 13. Discordant aneuploidies in monochorionic twins are rare. Aetiologies include mitotic error in early cell division and “rescue” chromosome loss in an initially trisomic zygote. Clinicians should offer early amniocentesis of both sacs and consider selective termination.

## Graphical abstract

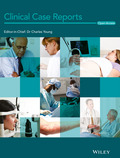

## 1 INTRODUCTION

The frequency of multiple pregnancy has risen rapidly in the era of advancing maternal age and increasing availability of assisted reproductive technologies. While all twin pregnancies carry higher risks of congenital abnormalities and perinatal mortality, monochorionic twins present unique challenges derived from their dependence on a shared placental circulation. The associated risks of twin‐to‐twin transfusion syndrome, selective intrauterine growth restriction, and twin‐anemia polycythaemia sequence result in the majority of perinatal deaths in these pregnancies^1,2^. Even with modern invasive fetal therapies, only 85% of monochorionic twin pregnancies result in two surviving twins.^1^

Although monochorionic twins have traditionally been assumed to be genetically identical, rare cases of heterokaryotypic monochorionic twins have been documented over the last three decades. Most articles describe discordance for either fetal sex or sex chromosome aneuploidy, particularly Turner's syndrome, but reports of autosomal aneuploidy (including trisomy 13, 18, and 21) are also starting to emerge^3^. However, despite accumulating reports, there are no consensus statements on how to manage monochorionic twins with discordant fetal abnormalities: patients and doctors are therefore faced with challenging uncertainty when they arise.

Although heterokaryotypic abnormalities are rare, when discordant anomalies and markers of aneuploidy are identified in the first or early second trimester, the merits and risks of chorionic villus sampling vs. double amniocentesis at 15‐16 weeks should be discussed; when both sacs are sampled, the individual karyotypes can be determined with certainty.^2^ In this article, we present a case of spontaneously conceived monochorionic diamniotic twins discordant for trisomy 13 and use a systematic literature search to collate management and outcome data from previously published similar reports. We highlight the important role of double amniocentesis in confirming the suspicion of discordant aneuploidy in monochorionic twins, demonstrate the utility of selective termination, and propose potential underlying genetic aetiologies. Written informed consent for publication was obtained from the patient.

## 2 CASE REPORT

This 26‐year‐old with previous medical termination of pregnancy at 22 weeks’ gestation for hypoplastic left heart syndrome was referred to our Fetal Medicine Unit in her second pregnancy. Dating ultrasound scan at 12 + 6 revealed monochorionic diamniotic twins with normal nuchal translucency (1.3 mm) and anatomy in Twin A but raised nuchal translucency (6.0 mm), generalized cutaneous edema and suspected encephalocele in Twin B. The patient was counseled on management options including expectant management, invasive testing, termination of the pregnancy, or selective termination of the affected twin. Findings for Twin B were confirmed on repeat ultrasound scans at 13 + 6 and 14 + 6, with multiple additional abnormalities identified that were suspicious for trisomy 13: semilobar holoprosencephaly, spina bifida, pulmonary atresia, ventricular septal defect, echogenic kidneys, and midline facial cleft. Management options were rediscussed at each scan and the patient eventually opted for selective termination of Twin B without prior invasive testing. This was performed uneventfully with radiofrequency cord ablation at 15 + 5 weeks. Microarray and karyotyping on amniotic fluid from Twin B revealed nondisjunctional trisomy 13.

Following additional counseling, the couple decided against genetic referral or further invasive testing. Close ultrasound surveillance of surviving Twin A confirmed normal anatomy, biometry and fetal echocardiogram, and fetal brain MRI was normal at 28 weeks. A 3470 g male infant was delivered in good condition by category 2 Cesarean section for failure to progress following induction of labor at 39 weeks and placental histopathology confirmed monochorionicity. Peripheral blood karyotyping of the surviving twin showed a normal male karyotype. Zygosity studies using a comparison of 16 short tandem repeats on chromosomes 13, 18, and 21 between the peripheral blood of Twin A and the amniocentesis sample from Twin B demonstrated that the samples were identical for all markers analyzed except for an increased chromosome 13 dosage in the amniocentesis sample, confirming discordance for trisomy 13. The surviving twin had an uncomplicated neonatal course and normal growth and neuro developmental follow‐up at the age of 2 years.

## 3 SEARCH STRATEGY

The search strategy (current to 08 August 2020) used text‐word variations and thesaurus terms for “monochorionic twins,” “trisomy 13,” and “discordance.” The databases searched were EMBASE and MEDLINE, with no language restrictions. Bibliographies of identified articles and conference abstracts from the Fetal Medicine Foundation and the International Society of Ultrasound in Obstetrics and Gynaecology were hand‐searched for eligibility. The search identified fourteen eligible articles. Five were excluded, due to: duplicates (1); articles describing monochorionic twins with discordant structural anomalies but identical karyotypes (2); and articles describing discordant aneuploidies other than trisomy 13 (2). Nine remaining articles^3^, ^4^, ^5^, ^6^, ^7^, ^8^, ^9^, ^10^, ^11^ were selected for full‐text review, all of which were included in the final analysis. These are summarized in Table 1.

**Table 1:** Case reports describing monochorionic twins discordant for trisomy 13

Author and year	Maternal age	Fetuses	Amniocentesis		Selective termination of affected twin		Outcome	Zygosity studies
			Yes/no	Karyotype result	Yes/no	Timing and method		
Heydanus et al., 1993^4^	Not reported	1 MCDA pair	No		No—abnormalities only detected at 25 weeks		IUFD of twin A (850 g, postnatal karyotype showed T13) and spontaneous preterm delivery at 27/40. NND of twin B (1060 g, postnatal karyotype normal)	Not reported
Lewi et al., 2006^5^	40	1 MCDA pair	Yes, both sacs	T13 in twin B only	Yes	Bipolar cord coagulation at 17 + 0	Live birth of surviving twin A at 40/40 (2580 g) with normal neuro‐developmental follow‐up	Not reported
Taylor et al., 2008^10^	40	DCTA triplets: 1 MCDA pair + 1 singleton	Yes, all three sacs	T13 in twin B only	Yes	Laser cord coagulation at 16 + 4	IUFD of cotwin at 18 + 4/40. Live birth of surviving singleton by elective Cesarean section at 36/40 (2722 g)	Monozygosity assumed (given this was an IVF conception with a two‐embryo transfer) but not confirmed
Sepulveda et al., 2010^8^	39	1 MCDA pair	Yes, both sacs	T13 in twin A only	No—illegal in Chile		PPROM at 31/40 and spontaneous preterm delivery at 32/40. Early NND of affected twin A (1050 g); unaffected twin B survived (1520 g) with normal neuro‐developmental follow‐up at age 12 mo	Monozygosity confirmed
Ramsey et al., 2012^7^	23	1 MCDA pair	Yes, both sacs	T13 in twin A only	No—offered but declined		Delivered by emergency Cesarean section at 32/40 due to FGR and nonreassuring CTG in the unaffected twin B. NND of affected twin A on day 6. Unaffected twin B survived with normal neuro‐developmental follow‐up at age 6 mo. Postnatal peripheral blood karyotype of the surviving twin showed mosaic trisomy 13 (5 of 50 cells examined)	Monozygosity confirmed
Dixit et al., 2012^3^	39	1 MCDA pair	Yes, both sacs	T13 in twin B only	Yes	Radiofrequency cord ablation at 18 + 0	Live birth of surviving twin A at 39 + 4/40 (3940 g) following induction of labor. Normal neuro‐developmental follow‐up at age 12 mo	Monozygosity confirmed
Spacek et al., 2015^9^	23	1 MCDA pair	Yes, both sacs	T13 in twin A only	Yes	Bipolar cord occlusion at 21 + 0	Live birth of surviving twin B by emergency Cesarean section at 36/40 due to FGR, oligohydramnios, and breech presentation (2400 g)	Monozygosity confirmed
McFadden et al., 2017^6^	29	1 MCDA pair	Yes, both sacs	T13 in twin A only	Yes	Radiofrequency cord ablation at 23 + 0	PPROM at 31 + 2/40 and spontaneous preterm delivery of surviving twin B at 32 + 2/40. Postnatal peripheral blood karyotype showed 15% mosaic trisomy 13, although skin fibroblast karyotype was normal	Not reported
Vojtěch et al., 2017^11^	Details not available	1 MCDA pair	Details not available	T13 in twin A only	Yes	Bipolar cord occlusion, gestation unknown	Live birth of unaffected twin: details not available on gestational age at delivery or neuro‐developmental follow‐up	Details not available
Current case	26	1 MCDA pair	No; proceeded straight to selective termination; amniocentesis of twin B only done at the time of termination	T13 in twin B	Yes	Radiofrequency cord ablation at 15 + 5	Live birth of surviving twin A by category 2 emergency Cesarean section at 39 + 3/40 for failure to progress following induction of labor (3470 g). Normal growth and development at age 24 months	Monozygosity confirmed

Abbreviations: CTG, cardiotocograph; FGR, fetal growth restriction; IUFD, intrauterine fetal death; MCDA, monochorionic diamniotic; NND, neonatal death; PPROM, preterm prelabor rupture of membranes; T13, trisomy 13.

## 4 DISCUSSION

This unusual case is the tenth reported incidence of monochorionic twins discordant for trisomy 13. Of note, the case reported by Taylor et al.^10^ features a dichorionic triamniotic triplet pregnancy conceived through in vitro fertilization with two‐embryo transfer: this article was eligible for inclusion in our analysis because the monochorionic twin pair was found to be discordant for trisomy 13. Details of the case reported by Vojtěch et al^11^ are incomplete because we were unable to access the full‐text article despite contacting the authors.

In the first case, published by Heydanus et al. in,^4^ discordant structural anomalies were identified on ultrasound at 25 weeks. The affected Twin A subsequently died in utero and after delivery at 27 weeks, Twin B died in the early neonatal period. Although no invasive antenatal testing was done, postnatal karyotyping confirmed trisomy 13 in the affected twin but normal chromosome complement in Twin B^2^. In at least seven of the subsequent nine cases^3‐9^ (details unavailable from Vojtěch et al,^11^ amniocentesis of both sacs was performed (at gestational ages ranging from 13 + 6 to 23 weeks) and confirmed discordant karyotypes, with one twin affected by trisomy 13 and the other with normal karyotype. Uniquely in this series, our patient decided against amniocentesis prior to selective reduction: given the obvious severity of the affected twin's abnormalities, the result would not have altered her decision for selective termination but would have risked miscarriage of the unaffected twin. In addition, the normal sonographic appearances of Twin A provided relative reassurance both before and after the diagnosis of trisomy 13 in Twin B.

Selective termination was performed in seven of the ten cases, at gestational ages ranging from 15 + 5 to 23 + 0 weeks: three with radiofrequency ablation, three with bipolar cord occlusion, and one with laser cord coagulation. In the case of laser cord coagulation, the unaffected cotwin subsequently died at 18 + 4.^10^ In the other six cases, the unaffected cotwin survived,gestational age at delivery ranged from 32 + 2 to 40 weeks, with only one unaffected cotwin delivered before 36 weeks.^6^

Three cases were managed expectantly (one only diagnosed at 25 weeks so termination not offered,^4^ one case from Chile where termination is illegal under all circumstances,^8^ and one in which termination was offered but declined.^7^ One of these resulted in loss of both twins as discussed above,^4^ the other two both resulted in delivery at 32 weeks with neonatal death of the affected twin and survival of the unaffected cotwin.^7^, ^8^

As discussed by the cited authors, there are several potential mechanisms for discordant karyotypes in monochorionic twins. These include:

1. Dizygosity, in which monochorionic twins are assumed to be monozygotic but in fact result from dizygotic conception with early fusion of the outer cell mass.^5^, ^7^
2. Mitotic error during an early postzygotic cell division, leading to aneuploidy in that cell lineage.^3^, ^5^, ^6^, ^7^, ^9^
3. Spontaneous “rescue” chromosome loss in an initially trisomic zygote.^3^, ^5^, ^6^, ^7^, ^8^

Both mitotic error and trisomic rescue can give rise to uniparental disomy and mosaicism.^3^, ^5^, ^6^, ^7^, ^8^, ^10^ Uniparental disomy may lead to phenotypic abnormalities if the chromosome involved has a high proportion of imprinted genes, but as noted by Ramsey et al, four cases of paternal and one case of maternal uniparental disomy for chromosome 13 have been reported, all with normal phenotypes.^7^ Similarly, mosaicism in the surviving twin may arise due to transplacental transfer of trisomic cells from the affected twin, as seen in the cases reported by Ramsey et al and McFadden et al^6^, ^7^ The possibility of mosaicism warrants postnatal karyotyping and close follow‐up of the structurally normal twin in a discordant pair.

## 5 CONCLUSION

This article reports the tenth case of monochorionic twins discordant for trisomy 13 and the first systematic synthesis of all previously published reports. Despite the obvious limitations of this analysis (including the small numbers, the variety of techniques used for selective termination and the fact that monozygosity was not always confirmed), it is likely that future similar cases will arise as rates of monochorionic twin conceptions and early detection of structural abnormalities increase, making this scenario increasingly relevant. Taken together, these ten cases support the use of selective termination and demonstrate good survival rates in the unaffected cotwin: 80% overall (8/10); 85.7% with selective termination (6/7). While all previous reports have advocated early amniocentesis of both sacs (which remains the standard management in these cases), they have not explicitly acknowledged the complexities of this decision for the parents, including the inherent risk of miscarriage, the psychological burden of additional waits for amniocentesis results and the likely increased risk to the cotwin from later termination.

## CONFLICT OF INTEREST

The authors declare no conflicts of interest.

## AUTHOR CONTRIBUTIONS

EFC: conceived the article and wrote the first draft of the manuscript. RW and GA: provided critical revisions of the manuscript and edited the text. All authors contributed to manuscript revision and read and approved the submitted version.

## EDITORIAL POLICIES AND ETHICAL CONSIDERATIONS

Written informed consent for publication obtained.
